# Introductory editorial for the thematic collection “blood purification in sepsis: from bench to bedside” in intensive care medicine experimental

**DOI:** 10.1186/s40635-023-00555-x

**Published:** 2023-09-30

**Authors:** Klaus Stahl, Sascha David

**Affiliations:** 1 Department of Gastroenterology, Hepatology, Infectious Diseases and Endocrinology, Hannover Medical School, Carl-Neuberg Straße 1, 30163 Hannover, Germany; 2 Institute of Intensive Care Medicine, University Hospital Zurich, Zurich, Switzerland; 3 Department of Nephrology, Hannover Medical School, Hannover, Germany

**Keywords:** Sepsis, Septic shock, Blood purification, Hemofiltration, Hemoadsorption, Plasmapheresis, Current status, Research agenda

Patients with septic shock do usually not die from the infection per se, but rather from their uncontrolled host response to it [1]. Despite the improved understanding of central pathophysiological mechanisms, the current treatment arsenal in septic shock is still restricted to infection control and supportive measures, such as circulatory support and organ replacement therapies [2]. Even if these principles are certainly essential to keep the patient alive, they do not represent a specific *sepsis therapy*, that would instead have to modulate the pathological host response itself, consisting of immune alterations, endothelial dysfunction and coagulopathy [1]. Unfortunately, multiple therapeutic approaches that can cure mice, based primarily on modulating *singular* sepsis mediators, have failed to show any survival benefit in humans. While the reasons for this are certainly manifold [3], doubt remains as to whether the modification of a single component in a highly complex network can actually lead to a relevant improvement in outcome.

Thus, it is not surprising that the idea of extracorporeal blood purification (aside from classical renal replacement therapy) to eliminate injurious mediators of the sepsis syndrome has received increasing interest over the last years. In fact, blood purification techniques, such as hemoadsorption, are already widely used in clinical practice due to its plausible theoretical rationale, despite lack of clear evidence. Moreover, some recent studies have even raised important risk–benefit concerns [4, 5] underlining the fundamental need of further research in this field.

Are we missing something important here? Is our understanding of the mechanisms, by which blood purification in sepsis might bring benefit or harm to the patient, still too limited to consider its widespread use in daily clinical practice? What is our current clinical practice in considering blood purification for patients with sepsis and septic shock? Are there other modes or sequences of blood purification that might enable a rebalancing approach of sepsis pathophysiology instead of pure removal of mediators? Which patients might benefit and which patients might not by the use of blood purification? How should we proceed to best explore these open questions in pre-clinical investigations as well as future clinical trials?

This thematic collection invites authors to focus on both pre-clinical and clinical approaches to explore future perspectives of blood purification in the additive treatment of sepsis. The aim is not only to contribute to an improved understanding of the molecular mechanisms influenced by blood purification but also to set the stage for taking the next steps to improve clinical management.

## Data Availability

Not applicable.
